# Macrolide-Resistant Mycoplasma pneumoniae Infection Successfully Treated With Doxycycline and Immunomodulators in a Pediatric Patient: A Case Report

**DOI:** 10.7759/cureus.92977

**Published:** 2025-09-22

**Authors:** Mekdes M Wollel, Muluneh A Yimer, Kathy Yimer

**Affiliations:** 1 Pediatrics, Rainbow Kids Urgent Care, Folsom, USA

**Keywords:** corticosteroids, doxycycline, immunomodulators, macrolide-resistant, mycoplasma pneumoniae, pediatric pneumonia, resistance genes, treatment failure

## Abstract

Mycoplasma pneumoniae (MP) is a common cause of community-acquired pneumonia in children and usually responds well to macrolide antibiotics. However, in recent years, the emergence of macrolide-resistant Mycoplasma pneumoniae (MRMP) is becoming a significant concern in some areas of the world, particularly in Asia, though it remains rare and not commonly reported in the United States.

We report a case of a previously healthy early teenage boy presenting with persistent dry cough, chest tightness, sore throat, headache, fatigue, and fever. Despite completing a full course of azithromycin, his symptoms persisted, and radiographic findings showed no improvement. Repeat multiplex polymerase chain reaction(PCR) confirmed ongoing MP infection, and resistance genes were identified. The patient was subsequently treated with doxycycline, along with systemic corticosteroids and bronchodilators, which led to rapid clinical and radiologic improvement within 72 hours of doxycycline initiation.

This case highlights the importance of considering MRMP in patients with confirmed MP infection who fail to respond to macrolide therapy, even in areas with low prevalence of mycoplasma infection. Despite the presence of atypical resistance genes in our case, the patient’s clinical course strongly supported the diagnosis of MRMP. It also underscores the potential role of adjunctive immunomodulator therapies, such as corticosteroids, alongside antibiotics in managing airway inflammation and bronchospasm associated with MP infection. Early diagnosis, appropriate antibiotics, and the use of immunomodulators are critical for achieving a fast recovery from the illness.

## Introduction

Mycoplasma pneumonia (MP) is a common cause of community-acquired pneumonia in children and young adolescents, accounting for up to 40% of cases [1]. In the majority of patients, it causes mild, self-limited illnesses that respond well to macrolide antibiotics such as azithromycin, which are widely used in pediatric patients due to their safety. Unlike many other bacterial pneumonias, MP cannot be treated with beta-lactam antibiotics because it lacks a cell wall, making macrolides the preferred first-line therapy. Over the past two decades, macrolide-resistant Mycoplasma pneumoniae (MRMP) has increased globally, reaching rates as high as 90% particularly in parts of Asia, while remaining below 10% in Europe and the United States [2, 3]. In the United States, documented cases of MRMP in children remain uncommon and are rarely reported, which may contribute to delayed diagnosis and inadequate treatment of the case.

Macrolide resistance is clinically concerning since it leads to treatment failure, prolongs patient recovery, and increases risks of complications. In children who failed to improve with macrolides, the limited availability of rapid resistance testing and the need for age-appropriate alternative antibiotics make treatment decisions difficult. While fluoroquinolones and tetracyclines are effective against resistant strains, their use is restricted in younger children [4] due to side effects. Doxycycline may be considered in children over eight years of age when resistance is suspected or confirmed.

Here, we report a case of MRMP infection in a 13-year-old boy in California. The patient did not respond despite a full course of macrolide therapy but showed rapid clinical improvement after starting doxycycline with corticosteroids, used as an immunomodulatory therapy to reduce inflammation. This case underscores the importance of considering MRMP even in regions with low prevalence and highlights the role of adjunctive immunomodulatory treatment in achieving favorable outcomes.

## Case presentation

A 13-year-old boy with no significant past medical history presented to urgent care with a three-day history of dry, persistent cough and chest tightness, accompanied by sore throat, fatigue, and fever up to 101°F. He has no known history of contact with sick people, no history of recent travel, and was up to date on vaccinations.

**Figure 1:**
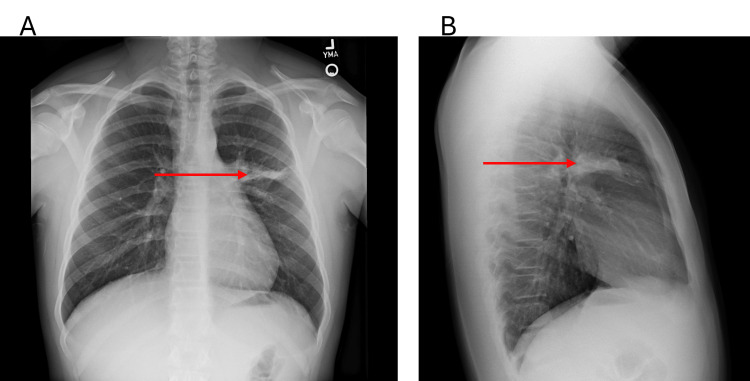
Posteroanterior (A) and lateral (B) chest X-rays at initial visit. Show left upper lobe consolidation at the initial visit. Arrows highlight areas of consolidation.

Upon initial presentation, physical examination findings were as follows: blood pressure (BP) 146/77 mmHg noted as elevated for age (>99th percentile), heart rate 95 beats per minute, respiratory rate 24 breaths per minute, temperature 99.8 °F (oral), oxygen saturation 97% on room air. His weight was 74.1 kg (163 lb. 5.8 oz), height 176.5 cm, body mass index 23.79 kg/m². He appeared sick but was non-toxic and was not in distress. Examination revealed mild posterior oropharyngeal erythema without exudates. There was no oral or perioral cyanosis and no nasal discharge. Lungs were clear with no abnormal sounds heard. No other significant abnormalities were detected in other systems. A chest X-ray showed left upper lobe consolidation (Figures 1A, 1B), and a multiplex polymerase chain reaction (PCR) (Spotfire) from a pharyngeal swab was positive for MP (Table 1). The diagnosis of atypical pneumonia (walking pneumonia) secondary to MP, accompanied by chest tightness, was considered likely to be due to bronchospasm resulting from MP. The patient was started on azithromycin 500mg on day one, then 250mg daily for five days. Albuterol metered dose inhaler (MDI) as needed for chest tightness, and supportive care including hydration, rest, and acetaminophen (Tylenol) as needed. Elevated BP might be related to illness or stress, but outpatient follow-up is recommended. With these interventions, the patient initially showed improvement, including resolution of fever, then symptoms plateaued without full resolution, and progressively worsened.

**Table 1:** Multiplex PCR results from pharyngeal swabs during first and third visit. PCR: polymerase chain reaction.

Bacteria	Result	Viruses	Result
Chlamydia pneumoniae	Negative	Adenovirus	Negative
Mycoplasma pneumoniae	Positive	Corona virus (seasonal)	Negative
Streptococcus dysgalactiae	Negative	Human metapneumovirus	Negative
Streptococcus pyogenes (group A strep)	Negative	Human rhinovirus/enterovirus	Negative
		Influenza A virus	Negative
		Influenza A virus A/H1-2009	Negative
		Influenza A virus A/H3	Negative
		Influenza B virus	Negative
		Parainfluenza virus	Negative
		Respiratory syncytial Virus	Negative

Second visit (day 10)

**Figure 2:**
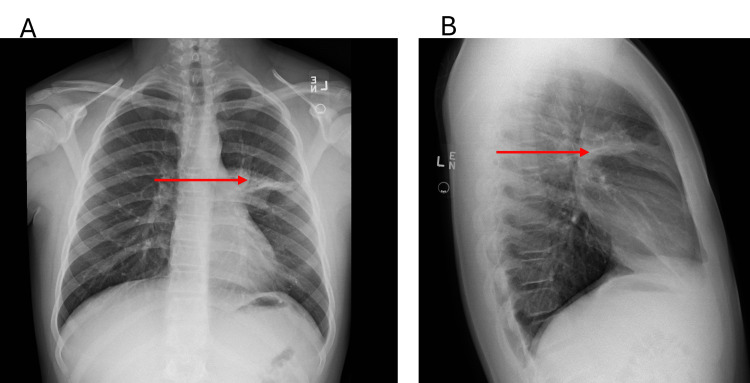
Posteroanterior (A) and lateral (B) chest X-rays on the second visit. A and B show persistent consolidation despite initial therapy on the second visit. Arrows highlight areas of consolidation.

Despite completing the course of azithromycin and all recommended interventions on the initial visit, the patient did not show full clinical or radiologic improvement. Ten days after the initial presentation, he returned to urgent care with a persistent cough, chest tightness, and a new onset of localized upper back pain without any history of trauma. He was afebrile, and all other vital signs were stable; oxygen saturation remained at 97% on room air. On physical examination, the chest was resonant on percussion and clear on auscultation. The musculoskeletal examination was notable for musculoskeletal spasms and tenderness in the left upper thoracic back, as well as reproducible pain. No other abnormalities were detected in the remaining systems. Repeated chest X-ray showed persistent left upper lobe infiltrate consistent with prior pneumonia (Figures 2A, 2B). By considering possible co-infection with typical pathogens such as Streptococcus pneumonia, in addition to MP, amoxicillin 500mg orally was empirically initiated twice a day to broaden coverage for typical pathogens. This decision was made before the availability of gene resistance testing. Supportive care with adequate hydration and symptomatic management was continued. However, after 48 hours of amoxicillin therapy, there was no discernible improvement in the patient's overall clinical condition.

Third visit (day 13)

After three days of amoxicillin therapy, the patient returned for a third time with a worsening cough, persistent chest tightness, and a new-onset frontal headache that persists throughout the day, but no associated symptoms of vomiting, blurring of vision, vertigo, or other neurological danger signs. On examination, he was tachypneic with a respiratory rate of 24 breaths per minute, decreased air movement, and bilateral wheezing, more pronounced on the left, accompanied by rales. Duoneb (albuterol/ipratropium) nebulizer treatment trial improved his symptoms and oxygen saturation. Oral dexamethasone (10mg) was given to mitigate the inflammatory bronchospasm. Repeat multiplex PCR remained positive for MP, with the rest of the panel negative for common respiratory viruses (Table 1). Gene resistance testing confirmed macrolide resistance genes (Table 2), and the molecular pathology report from pharyngeal swab showed detection of Staphylococcus aureus, albeit with low bacterial load. Low bacterial load suggests contamination from skin flora during sampling rather than active infection. The activity of different antibiotics for the detected Staphylococcus aureus bacteria is given in Table 3.

**Table 2:** Gene resistance test results from pharyngeal swab. Resistance genes detected and associated resistance mechanism. ermB/ermC: macrolide–lincosamide–streptogramin resistance; mefA: macrolide efflux pump; tetB/tetM: tetracycline resistance.

Resistance Genes	Antibiotics	Result
ermB, C; mefA	Potential resistance to the macrolide, lincosamide and streptogramin	Detected
tet B, tet M	Potential resistance to tetracycline, doxycycline and minocycline	Detected

**Table 3:** Antibiotic activity against the detected Staphylococcus aureus. DHFR: dihydrofolate reductase, (++) = best activity, (+) = good activity and (±) = variable activity.

Antibiotic Class	Antibiotic (Route)	Activity vs S. aureus
Penicillins	Cloxacillin (po)	++
	Dicloxacillin (po)	++
	Penicillin VK (po)	±
	Ampicillin (IV/IM/po)	±
	Amoxicillin (po)	±
	Amoxicillin Clavulanic acid (po)	+
	Delafloxacin (po/IV)	+
Fluoroquinolones	Ofloxacin (po/OT/OP)	+
	Levofloxacin (po/OP/IV)	+
	Moxifloxacin (po/OP/IV)	+
	Gemifloxacin (po)	+
	Cefuroxime (po/IV/IM)	+
Parenteral Cephalosporins	Cefadroxil (po)	+
Oral Cephalosporins	Cephalexin (po)	+
	Cefaclor (po)	+
	Cefprozil (po)	+
	Cefuroxime Axetil (po/IV/IM)	+
	Cefpodoxime (po)	+
	Cefdinir (po)	+
	Cefditoren (po)	+
	Omadacycline (po/IV)	+
Tetracyclines	Vancomycin (po/IV/OP)	+
Glycopeptides/Lipoglycopeptide	Linezolid (po/IV)	+
Oxazolidinones	Tedizolid (po/IV)	+
	Lefamulin (po/IV)	+
Pleuromutilin	Trimethoprim Sulfamethoxazole (Bactrim, po/IV)	+
DHFR inhibitor/sulfonamide	Trimethoprim Sulfamethoxazole (Bactrim, po/IV)	+

Final management and outcome

Given the patient’s lack of improvement with both azithromycin and amoxicillin, persistent radiographic findings, and genetic confirmation of MRMP, the diagnosis of community-acquired pneumonia secondary to MRMP with bronchospasm was established. He was started on doxycycline, 100mg twice daily, to target the resistant pathogen. Dexamethasone 10mg PO daily was continued for two additional days, in conjunction with albuterol MDI as needed for wheezing and chest tightness, likely due to inflammatory bronchospasm.

**Figure 3:**
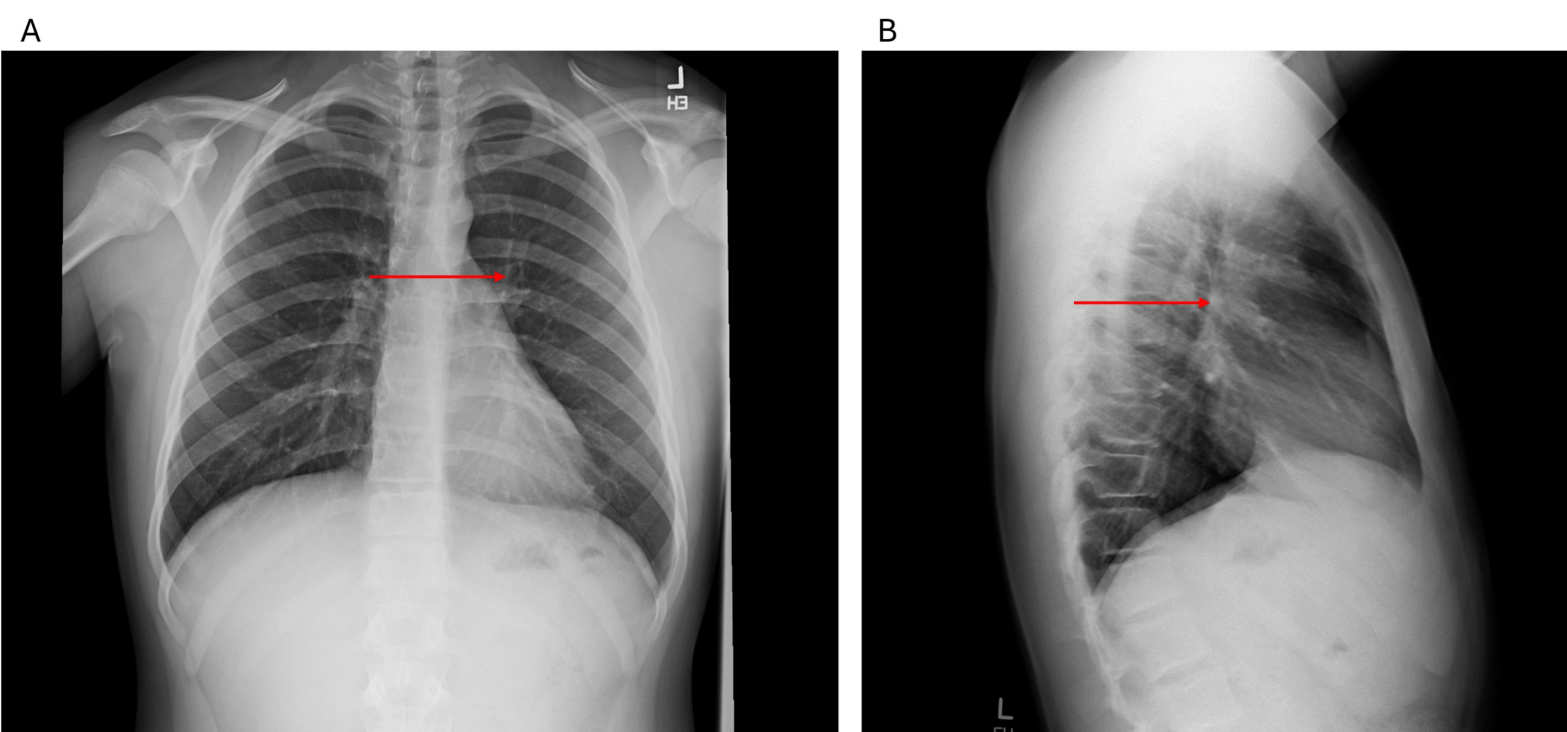
Follow-up posteroanterior (A) and lateral (B) chest X-ray after recovery. Arrows indicate previously affected areas now resolved.

Within 72 hours of initiating doxycycline, the patient began to show clinical improvement. On follow-up evaluation after completing the full course of doxycycline, he demonstrated marked clinical recovery, and a follow-up chest X-ray also showed significant radiologic resolution (Figure 3A, 3B). The clinical timeline showing symptoms, imaging, diagnostics, treatment, and outcomes is summarized in Table 4.

**Table 4:** Clinical timeline summarizing symptoms, imaging, diagnostics, treatment, and outcomes. CXR: chest X-ray, LUL: left upper lobe, PCR: polymerase chain reaction.

Day	Symptoms	Imaging	PCR / Resistance	Treatment	Outcome
3 (Initial)	Dry cough, chest tightness, sore throat, fever	CXR: LUL consolidation	PCR + M. pneumoniae	Azithromycin ×5d, albuterol, supportive care	Fever resolved, cough persistent
10	Persistent cough, chest tightness, new back pain	CXR: persistent consolidation	PCR + M. pneumoniae	Amoxicillin empirically added	No improvement
13	Worsening cough, chest tightness, headache, wheezing	CXR: persistent consolidation	PCR + M. pneumoniae, macrolide resistance confirmed	Doxycycline ×10d, dexamethasone ×2d, albuterol	Rapid improvement
Follow-up after one week	Symptom resolution	CXR: resolution of consolidation	N/A	Completed doxycycline course	Full recovery

## Discussion

MP is a small, slow-growing bacterium first isolated in 1944 from a patient with atypical pneumonia [4]. It is a common cause of atypical pneumonia in children, adolescents, and adults, primarily transmitted through respiratory droplets from person to person. Most patients with MP infection present with mild, non-specific symptoms, including cough, fever, malaise, sore throat, and wheezing. Only approximately 0.5-2% of cases progress to severe forms of intrapulmonary and extrapulmonary manifestations [5]. The pathogenesis of MPP is associated with both microbial virulence factors and a hyperresponsive host immune response to the infection. This exaggerated immune activation against the infection results in pulmonary inflammation and tissue injury, often contributing to more severe disease than the pathogen itself [6, 7].

Since mycoplasmas have no cell wall, they are intrinsically resistant to β-lactams and all other antibiotics that act on the cell wall. Macrolides are first-line antibiotics for treating Mycoplasma infections, particularly in pediatric populations. However, MRMP has increased worldwide nowadays.

The mechanism of action for macrolide antibiotics primarily involves decreasing protein synthesis by binding to the central ring of the 23S ribosomal RNA (rRNA) V region of the ribosomal 50S large subunit. A single base mutation causes macrolide resistance in MP, commonly A2063G, in the 23S rRNA gene, which leads to a structural change of the main binding site of the macrolide antibiotics and reduces the binding affinity of macrolides to the ribosomal subunit [8, 9]. The first documented case of MRMP was reported in Japan in 2001 [10]. After initial detection in Japan, MRMP cases increased globally, with the highest incidence in Asia but rarely reported in Europe and the United States.

In this report, we described MRMP infection in a child successfully treated with doxycycline, an immunomodulator, and a systemic corticosteroid. Initial diagnosis of atypical pneumonia due to MP was supported by symptoms consistent with atypical pneumonia, chest imaging, and a positive multiplex PCR for MP. With this diagnosis, he was started on standard treatment with azithromycin and subsequent empiric coverage with amoxicillin, Albuterol MDI, and other supportive management. However, despite all these interventions, the patient's persistent symptoms with new onset back pain and chest tightness, possibly from bronchospasm, lack of radiologic improvement, and repeated positive PCR raised concern for treatment failure. A gene resistance test showed macrolide resistance genes (ermB, ermC, and mefA) as well as tetracycline resistance genes (tet B, tet M) (Table 2).

Macrolide resistance genes ermB, ermC, and mefA are not commonly associated with MP [11-13]. However, some recent studies on MRMP in adult patients have reported the presence of efflux pump genes such as mefA and msrA/B in MP clinical isolates [14]. These genes (mefA and msrA/B) represent less common resistance mechanisms compared to 23S rRNA gene mutations, but they may still contribute partially to macrolide resistance.

In our case, although ermB and ermC are not typically linked to MP, the confirmed presence of the organism by multiplex PCR, the detection of the efflux pump gene mefA (despite its relatively rare role compared to 23S rRNA mutations), lack of clinical response to azithromycin, and marked improvement following doxycycline therapy strongly support the diagnosis of MRMP.

 The use of doxycycline in the management of MRMP in children under eight years is limited due to the side effects of teeth discoloration [4, 15], but it remains a preferable option in older children. In our case, since the patient is older than eight years, he started on doxycycline. The patient exhibited marked clinical and radiologic improvement following initiation of doxycycline. Chest tightness and wheezing in this patient were possibly mediated by immune hyperactivation and airway hyperresponsiveness from MP infection. The addition of immunomodulators and bronchodilators in our patient played an important role in symptom control, particularly in reducing wheezing and airway inflammation [6, 16].

The tetracycline resistance genes tetB and tetM, detected on resistance gene testing, are not typically associated with MP [17, 18]. Instead, these genes are more commonly found in gram-positive cocci, such as Streptococcus pneumoniae and Staphylococcus aureus [18-20]. So, in our patient, clear clinical improvement of the patient following doxycycline therapy despite the presence of these resistance genes and concurrent detection of low-load Staphylococcus aureus raises the possibility that these resistance genes were derived from contaminant Staphylococcus aureus rather than the Mycoplasma itself.

## Conclusions

Reports of MRMP remain uncommon in the United States, particularly in pediatric populations, compared to regions such as Asia, where resistance rates can reach up to 90%. We report a confirmed case of MRMP in a 13-year-old boy in California, identified through PCR resistance gene testing after inadequate clinical response to macrolide therapy. Despite the presence of atypical resistance genes, the patient demonstrated clear clinical and radiologic improvement following treatment with doxycycline. The addition of systemic corticosteroids and bronchodilators played a critical role in managing airway inflammation and controlling symptoms. We hope our case report could be a useful reference and clinical reminder for considering MRMP early in the differential diagnosis for patients with confirmed MP infection who fail to respond to standard macrolide treatment, even in regions with low resistance prevalence, and the potential role of immunomodulators such as steroids in the treatment of such cases. It also highlights that clinical judgment and treatment response remain critical in diagnosing MRMP, especially when atypical or non-classical resistance genes are identified, as in our case.
